# A bone overgrowth disorder due to a gain-of-function mutation in the kinase homology domain of guanylyl cyclase B, the receptor for CNP

**DOI:** 10.1186/2050-6511-14-S1-P35

**Published:** 2013-08-29

**Authors:** Michaela Kuhn, Thomas Premsler, Ruey-Bing Yang, Thomas D Mueller, Birgit Gaßner, Heike Oberwinkler, Sabine E Hannema, Hermine A van Duyvenvoorde, Ferdinand Roelfsema, Gijs WE Santen, Timothy Prickett, Sarina G Kant, Annemieke JMH Verkerk, André G Uitterlinden, Eric Espiner, Claudia AL Ruivenkamp, Wilma Oostdijk, Alberto M Pereira, Monique Losekoot, Jan M Wit

**Affiliations:** 1 Institute of Physiology, University of Würzburg, Germany; 2 Institute of Biomedical Sciences, Academia Sinica Taipei, Taiwan; 3 Department of Molecular Plant Physiology and Biophysics, Julius-von-Sachs-Institute, Biocenter, University of Würzburg, Germany; 4 Department of Paediatrics, Leiden University Medical Centre, Leiden, The Netherlands; 5 Department of Endocrinology and Metabolic Diseases, Leiden University Medical Centre, Leiden, The Netherlands; 6 Department of Clinical Genetics, Leiden University Medical Centre, Leiden, The Netherlands; 7 Department of Medicine, University of Otago, Christchurch New Zealand; 8 Department of Internal Medicine, Erasmus Medical Centre, Rotterdam, The Netherlands

## Background

C-type natriuretic peptide (CNP), via its guanylyl cyclase B (GC-B) receptor and intracellular cGMP, is critically involved in bone development by regulating growth plate chondrocyte differentiation and proliferation. Homozygous loss-of-function mutations in GC-B lead to short-limbed dwarfism (acromesomelic dysplasia, type Maroteaux). Here we describe a novel heterozygous gain-of-function mutation in an extremely tall patient displaying mild scoliosis and a non-Marfanoid habitus.

## Materials and methods

Whole exome sequencing revealed a heterozygous GC-B mutation resulting in a single amino acid exchange within the submembrane kinase homology domain (KHD). The impact on cGMP formation was studied in transfected HEK293 cells and in cultured fibroblasts obtained from the patient and healthy donors. The interaction of wildtype and mutated GC-B was evaluated by co-immunoprecipitation.

## Results

Basal and CNP-stimulated cGMP syntheses by homozygous and heterozygous mutant GC-B dimers were markedly increased in HEK293 cells and in patient skin fibroblasts. Homology modeling revealed that the mutation is adjacent to the ATP-binding pocket of the KHD domain. Notably, ATP potentiated CNP effects on wildtype and much more on mutated GC-B. Finally, co-IP demonstrated that wildtype und mutant GC-B form heterodimers, explaining the functional impact of this point mutation on receptor activity under (human) heterozygous conditions.

## Conclusion

Our study unravels for the first time a point mutation in the KHD of GC-B which dramatically enhances cGMP production by the adjacent GC domain. This remarks the regulatory role of the KHD and suggests that configuration of the ATP-binding pocket provides a critical allosteric regulatory step in CNP/GC-B signal transduction.

